# Brain Network Architecture and Plasticity: MR Neuroimaging Perspectives

**DOI:** 10.1155/2016/6952169

**Published:** 2016-03-27

**Authors:** Feng Shi, Yong Liu, Shuyu Li, Xiaobo Li, Martin Walter

**Affiliations:** ^1^Department of Radiology and Biomedical Research Imaging Center, University of North Carolina at Chapel Hill, Chapel Hill, NC 27599, USA; ^2^Brainnetome Center, Institute of Automation, Chinese Academy of Sciences, Beijing 100190, China; ^3^School of Biological Science and Medical Engineering, Beihang University, Beijing 100191, China; ^4^Department of Biomedical Engineering, New Jersey Institute of Technology, Newark, NJ 07102, USA; ^5^Eberhard Karls University, 72074 Tübingen, Germany

Recent advances in brain connectivity research suggest that the human brain operates as a complex but economic global network. Novel approaches from graph theory have been applied to a variety of neuroimaging studies and have achieved great success. However, the brain network architecture and plasticity patterns vary across different brain developmental periods, learning activities, and disease effects. Translation of descriptive imaging levels into clinical and other exploitation crucially depends on the potential to induce and monitor plastic changes in the network of interest. This special issue is intended to stimulate the continuing efforts in understanding the architecture and plasticity patterns of brain networks. It also aims to broaden the attention not only to the researchers who are currently working on these areas but to the general scientific audience who are interested in the brain structures and functions as well.

This special issue includes eleven papers that presented an up-to-date progress of MR neuroimaging methods on brain network architecture and plasticity investigation employing various neuroimaging modalities: structural MRI, diffusion MRI, and functional MRI. In these papers, there are five studies focused on brain diseases, including Alzheimer's disease, mild cognitive impairment, mild traumatic brain injury, and major depressive disorder. Two studies investigated the brain plasticity changes after visual or hearing deprivations. Also, there are two studies that assessed volunteers after mediation or cognitive training, and, finally, the remaining two studies are on the methodological development for brain network construction.

*Brain Diseases.* In the paper entitled “Abnormal Resting-State Functional Connectivity Strength in Mild Cognitive Impairment and Its Conversion to Alzheimer's Disease” by Y. Li et al., an investigation was performed for individuals diagnosed with mild cognitive impairment. With a 2-year follow-up, those individuals converted to Alzheimer's disease were compared with nonconverters for their alterations on functional connectivity strength and seed-based functional connections.

The paper entitled “Topological Properties of Large-Scale Cortical Networks Based on Multiple Morphological Features in Amnestic Mild Cognitive Impairment” by Q. Li et al. investigated the topological properties of cortical networks based on geometric measures (i.e., sulcal depth, curvature, and metric distortion) change in aMCI patients compared with normal controls.

The paper entitled “Multilevel Deficiency of White Matter Connectivity Networks in Alzheimer's Disease: A Diffusion MRI Study with DTI and HARDI Models” by T. Wang et al. conducted an evaluation of how the fiber tractography method could influence the construction of brain white matter network and eventually affect the extraction of brain network properties, as well as group comparison using Alzheimer's disease patients as examples.

In the paper entitled “Compensation through Functional Hyperconnectivity: A Longitudinal Connectome Assessment of Mild Traumatic Brain Injury” by A. Iraji et al., patients with mild traumatic brain injury were studied for possible brain functional alterations. Brain functional network was constructed through predefined seeds in a 358-landmark mask and a groupwise clustering algorithm was employed to identify general patterns of functional hyperconnectivity.

The paper entitled “Reorganization of Anatomical Connectome following Electroconvulsive Therapy in Major Depressive Disorder” by J. Zeng et al. presented a study for patients with major depressive disorder that were treated by electroconvulsive therapy. The plasticity of white matter pathway was evaluated.

*Sensory Deprivation.* In the paper entitled “Alterations of Regional Spontaneous Brain Activity and Gray Matter Volume in the Blind” by A. Jiang et al., authors investigated how the early blindness shapes the regional spontaneous brain activity and gray matter volume in visual areas compared to sighted controls. A correlation analysis is also performed between the onset age of blindness and the brain structure and functions.

In the paper entitled “Functional Reorganizations of Brain Network in Prelingually Deaf Adolescents” by W. Li et al., authors used resting-state fMRI to study prelingually deaf adolescents to access the possible brain network reorganizations due to their experience.

*Training Influence.* The paper entitled “State and Training Effects of Mindfulness Meditation on Brain Networks Reflect Neuronal Mechanisms of Its Antidepressant Effect” by C.-C. Yang et al. designed a longitudinal analysis investigating resting-state fMRI both before and after 40 days of meditation training. Differences in functional connectivity both between states (rest versus meditation) and between timepoints (before versus after training) were assessed and reveal functional specificity of plastic reconfiguration of key networks implied in MDD.

The paper entitled “The Exercising Brain: Changes in Functional Connectivity Induced by an Integrated Multimodal Cognitive and Whole-Body Coordination Training” by T. Demirakca et al. investigated the impact of “life kinetik” training on brain plasticity in terms of an increased functional connectivity during resting-state functional magnetic resonance imaging (rs-fMRI). The training is an integrated multimodal training that combines motor and cognitive aspects and challenges the brain by introducing new and unfamiliar coordinative tasks.

*Methodology Development.* The paper entitled “Closely Spaced MEG Source Localization and Functional Connectivity Analysis Using a New Prewhitening Invariance of Noise Space Algorithm” by J. Zhang et al. proposed a prewhitening invariance of noise space as a new method for localizing closely spaced and highly correlated cortical sources under real magnetoencephalography (MEG) noise, to facilitate the source-level functional connectivity analysis.

The paper entitled “Node Detection Using High-Dimensional Fuzzy Parcellation Applied to the Insular Cortex” by U. Vercelli et al. investigated a fuzzy parcellation scheme that partitions insular cortex into a number of regions based on the variances of their functional signals. Furthermore, the identified 12 clusters located in the insular cortex are found to best correlate with distinct brain areas that subserve different brain circuits/functions.

